# Expression and regulatory roles of lncRNAs in G-CIMP-low vs G-CIMP-high Glioma: an in-silico analysis

**DOI:** 10.1186/s12967-021-02844-z

**Published:** 2021-04-29

**Authors:** Indrani Datta, Houtan Noushmehr, Chaya Brodie, Laila M. Poisson

**Affiliations:** 1 Department of Public Health Sciences, Center for Bioinformatics, Henry Ford Health System, 1 Ford Place, 3C, Detroit, MI 48202 USA; 2 Department of Neurosurgery, Hermelin Brain Tumor Center, Henry Ford Cancer Institute, Henry Ford Health System, Detroit, USA

**Keywords:** Long non-coding RNAs, Glioma, G-CIMP subtypes

## Abstract

**Background:**

Clinically relevant glioma subtypes, such as the glioma-CpG island methylator phenotype (G-CIMP), have been defined by epigenetics. In this study, the role of long non-coding RNAs in association with the poor-prognosis G-CMIP-low phenotype and the good-prognosis G-CMIP-high phenotype was investigated. Functional associations of lncRNAs with mRNAs and miRNAs were examined to hypothesize influencing factors of the aggressive phenotype.

**Methods:**

RNA-seq data on 250 samples from TCGA’s Pan-Glioma study, quantified for lncRNA and mRNAs (GENCODE v28), were analyzed for differential expression between G-CIMP-low and G-CIMP-high phenotypes. Functional interpretation of the differential lncRNAs was performed by Ingenuity Pathway Analysis. Spearman rank order correlation estimates between lncRNA, miRNA, and mRNA nominated differential lncRNA with a likely miRNA sponge function.

**Results:**

We identified 4371 differentially expressed features (mRNA = 3705; lncRNA = 666; FDR ≤ 5%). From these, the protein-coding gene *TP53* was identified as an upstream regulator of differential lncRNAs PANDAR and PVT1 (p = 0.0237) and enrichment was detected in the “development of carcinoma” (p = 0.0176). Two lncRNAs (HCG11, PART1) were positively correlated with 342 mRNAs, and their correlation estimates diminish after adjusting for either of the target miRNAs: hsa-miR-490-3p, hsa-miR-129-5p. This suggests a likely sponge function for HCG11 and PART1.

**Conclusions:**

These findings identify differential lncRNAs with oncogenic features that are associated with G-CIMP phenotypes. Further investigation with controlled experiments is needed to confirm the molecular relationships.

**Supplementary Information:**

The online version contains supplementary material available at 10.1186/s12967-021-02844-z.

## Background

Glioma, a tumor of glial cells, is the most aggressive form of tumor of the central nervous system (CNS). Historically glioma has been described by histologic features and malignancy grading. Glioblastoma (GBM) is grade IV disease, typically with necrotic regions, conferring poor overall survival (15.5% at 2 years for adult GBM, 95% CI: 15.1%–15.9%) [1, 2]. Diffuse gliomas, astrocytoma and oligodendroglioma of grade 2 or 3, are characterized by varying degrees of aggressiveness and extensive infiltrative growth in the surrounding CNS parenchyma [1, 2]. Recently, the World Health Organization (WHO) added the presence of one of the recurrent point mutations in the isocitrate dehydrogenase genes (*IDH1* or *IDH2*) and co-deletion of chromosomal arms 1p/19q to the glioma diagnosis criteria [3]. Yet, even the refined molecular diagnosis classifications do not fully explain the heterogeneous clinical phenotypes of these tumors.

With recent advances in genomics, molecular subtypes are able to be further refined. In glioma, characterization of the epigenome by DNA methylation assay has been useful in the stratification and integration of molecular and phenotypic features [3]. One such sub-classification, known as the CpG island methylator phenotype (CIMP), is defined by genome-wide hypermethylation of CpG Islands (CGI) and was first defined in the context of colorectal cancer [4, 5]. The glioma-CIMP (G-CIMP) subtype was first described by Noushmehr et al. [6] in glioblastoma (GBM; Grade 4 glioma) and then in lower-grade gliomas (LGG; Grades 2, 3). Compared to G-CIMP negative tumors, several studies found that G-CIMP positive subtypes were typically associated with younger patients and with *IDH*-mutant gliomas without 1p/19q co-deletion [6, 7]. This *IDH*-mutant G-CIMP positive subtype has now been further refined into two distinct subgroups, G-CIMP-low (10% of *IDH*-mutant, 1p/19q intact tumor) and G-CIMP-high (90% of *IDH*-mutant, 1p/19q intact tumors), with ‘low’ and ‘high’ designations determined by a low or high degree of DNA methylation, respectively. As opposed to the characteristically higher survival rate of IDH-mutant glioma, G-CIMP-low tumors have survival rates that are closer to that of GBM. Even though the two G-CMIP subtypes have molecularly distinct methylation patterns, factors driving the difference in prognosis are yet unknown. It is assumed that DNA methylation patterns are associated with transcriptomic patterns, including non-coding RNAs. With recent advances in RNA sequencing various RNA species can be quantified (e.g., coding messenger RNA [mRNA], micro RNA [miRNA;22–24 bp], and long non-coding RNA [lncRNA, > 200 bp]). Among the RNA species, epigenetic regulators such as long non-coding RNA (lncRNA) have gained attention in recent years in cancer research.

LncRNAs are minimally 200-nucleotide RNA, with no known translational capacity. They have drawn attention due to their potential to regulate many cellular activities, as well as gene expression, in biological and pathological processes. Acting as cellular address codes, lncRNAs transfer proteins to their appropriate chromosomal location or fold them into higher order structures as target recognition for chromatin remodeling. In glioma, lncRNAs have been associated with oncogenesis and prognosis [8, 9], such as in the recent global analysis of lncRNAs in TCGA grade 2–4 gliomas that identified a panel of 64 lncRNAs associated with prognosis [10]. Among the specific lncRNAs studied, HOTAIR—a well-known, highly-expressed lncRNA in breast cancer [11]—has been associated with biogenesis and differentiation of gliomas [10]. TALNEC2, a lncRNA highly expressed in GBMs and with poor prognosis when silenced, inhibited cell proliferation and arrested the cells in the G1\S phase of the cell cycle in patient-derived glioma cell lines [12]. In addition, some newly discovered lncRNAs such as lncRNA ASLNC22381 and KIAA0495 [9] have been found in glioma tissue and cell lines.

Since each of the glioma subtypes is clinically distinct, understanding the role of associated epigenetic regulators could help to better distinguish between the groups. Differential epigenetic regulators may also identify biological differences underlying the phenotypes or suggest novel therapeutic targets. In this study we aimed to identify differentially expressed lncRNA between G-CIMP-high and G-CIMP-low glioma, using RNA sequencing data from the glioblastoma (GBM; Grade 4 glioma) and lower grade glioma (LGG; Grades 2 and 3) arms of The Cancer Genome Atlas (TCGA). As the functional roles of most lncRNAs are poorly understood, we evaluated lncRNA involvement by gene-set enrichment from an available functional knowledgebase. Finally, we integrated lncRNA, miRNA, and mRNA expression through correlation estimates to identify lncRNAs that may be affecting transcription level changes in relationship to miRNA by acting as a miRNA sponge.

## Results

### Sample details

A summary of the clinical and demographic data of the TCGA G-CMIP cohort is represented in Table 1. These cases are *IDH* mutant tumors, without 1p/19q co-deletion, by definition of the G-CMIP phenotype. As such, the majority of the cases were under 40 years old at diagnosis; most tumors had an astrocytomatous histology. Primary diagnosis for these tumors used the 2007 WHO diagnosis guidelines. The WHO 2016 diagnosis was inferred from WHO grading (2/3 = Astrocytoma; 4 = GBM) and molecular features (IDH mutant, without 1p/19q co-deletion).

**Table 1:** Patient characteristics for the 250 primary glioma diagnoses in this study

	G-CIMP High	G-CIMP Low
Number of Cases	234	16
Age		
Under 40 (%)	149 (63.7%)	10 (62.5%)
Over 40 (%)	80 (34.2%)	6 (37.5%)
Unknown (%)	5 (2.1%)	0 (0.0%)
Gender		
Male (%)	133 (56.8%)	7 (43.8%)
Female (%)	100 (42.7%)	9 (56.2%)
Unknown (%)	1 (0.4%)	0 (0.0%)
WHO 2007 Histology		
Oligodendroglioma	39 (16.7%)	0 (0.0%)
Oligoastrocytoma	74 (31.6%)	1 (6.2%)
Astrocytoma	118 (50.4%)	10 (62.5%)
Glioblastoma	2 (0.9%)	5 (31.2%)
WHO Grade		
2	111 (47.4%)	
3	92 (39.3%)	8 (50.0%)
4	2 (0.9%)	5 (31.2%)
WHO 2016 Diagnosis		
IDHmut – Astroctyoma	232 (99.1%)	11 (68.8%)
IDHmut – Glioblastoma	2 (0.9%)	5 (31.2%)

### Association of lncRNA expression with G-CIMP group

**Fig. 1:**
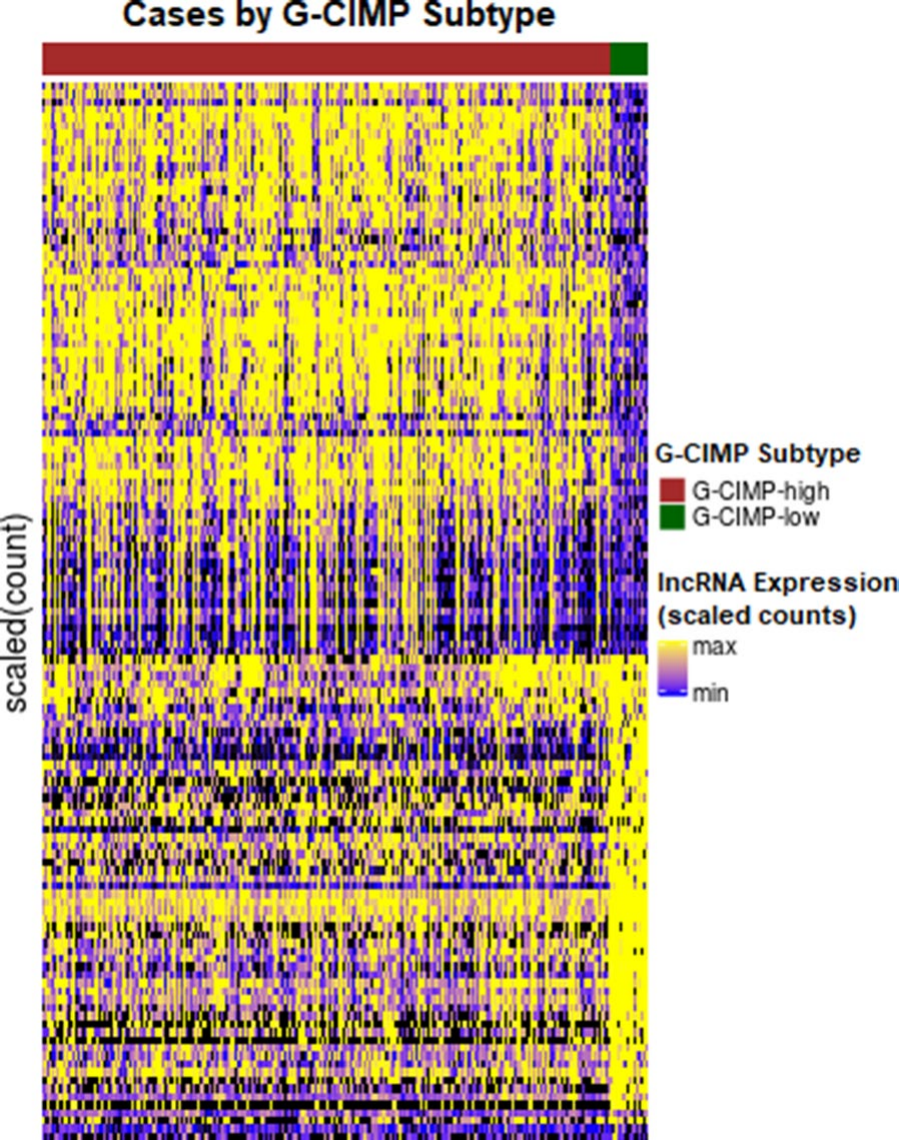
The heatmap of differentially expressed lncRNAs with a fold change of at least 2. Here the expression level is presented as standard deviations from the mean per lncRNA (row), with yellow high, blue low, and black at the mean. The lncRNAs (rows) are ordered by hierarchical clustering

The comparison of RNA sequencing reads between glioma subtypes G-CIMP-high and G-CIMP-low identified 4371 differentially expressed (DE) features (mRNA = 3705, lncRNA = 666) at a false discovery rate of 0.05. Figure 1 shows a heatmap of the DE lncRNAs between GCIMP-high and GCIMP-low groups. Here the expression level is standardized per row with yellow high and blue low. The rows are ordered by hierarchical clustering of lncRNA expression. The majority of the 666 differential lncRNAs identified are highly expressed, with a maximum fold change of expression in G-CIMP-low tumors four times that of G-CIMP-high tumors.

**Fig. 2:**
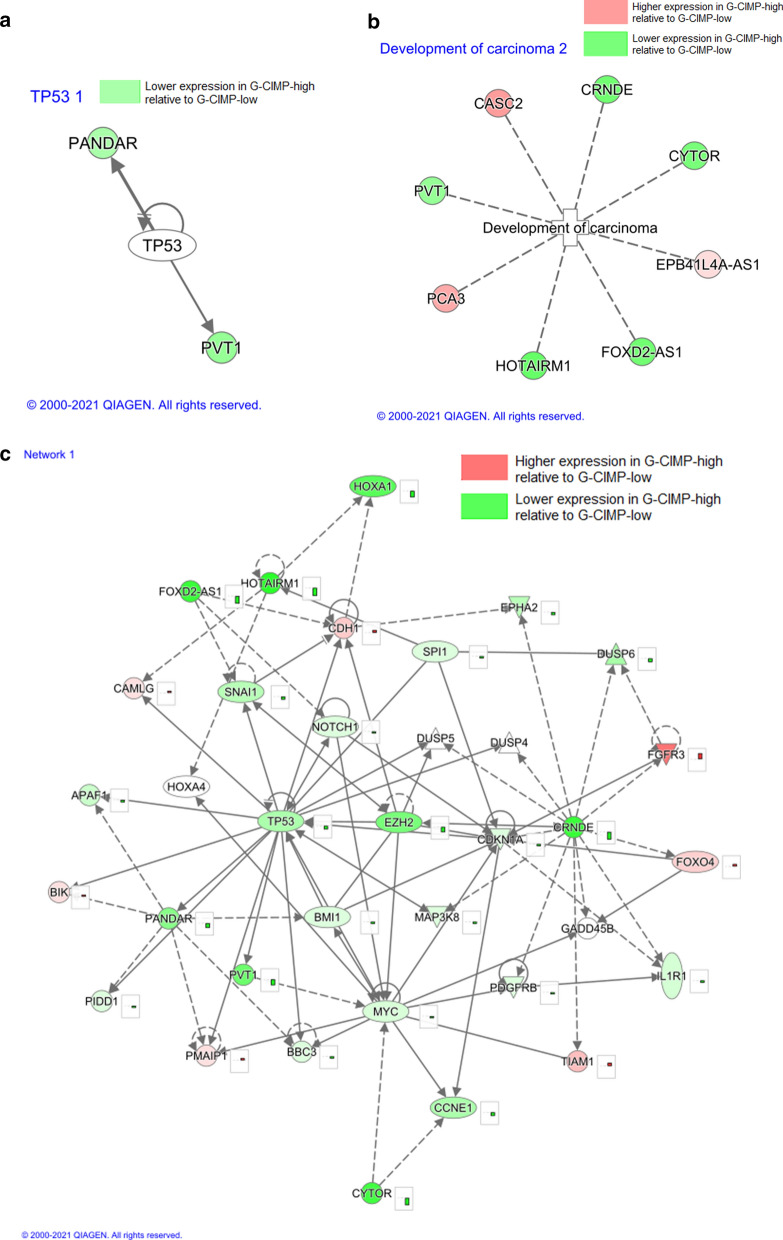
Results from Ingenuity Pathway Analysis, **a** TP53 was identified as an upstream regulator of PANDAR and PVT1. **b** Differential lncRNAs associated with development of a carcinogenic activity. **c** This gene–gene network includes 6 differentially expressed lncRNAs (PVT1, PANDAR, FOXD2-AS1, CYTOR, CRNDE, and HOTAIRM1) and captures elements of cell growth, proliferation, survival and death

The biological role of the DE lncRNA was explored via pathway analyses. Of the 666 DE lncRNAs, 44 were identified by lncRNA gene symbols in the QIAGEN Knowledge Base and therefore available for analysis by the Ingenuity Pathway Analysis tools. Set-enrichment analyses identified protein-coding gene *TP53* as an upstream regulator of DE lncRNA PANDAR and PVT1 (p = 0.0237; Fig. 2a). In addition, “development of carcinoma” was identified as an enriched disease category (Fig. 2b). Specifically, four oncology-related sets were among the most enriched disease and biological function categories (Table 2): ‘breast or colorectal cancer’ (9 DE lncRNAs, p = 0.0049), ‘development of digestive organ tumor’ (8 DE lncRNAs, p = 0.0122), ‘development of carcinoma’ (9 DE lncRNAs, p = 0.0176), and ‘malignant genitourinary solid tumor’ (8 DE lncRNAs, p = 0.0254). A top constructed biological network associated with the DE lncRNA list was associated with cell death and survival, cellular growth, and proliferation cellular development. This network was based on 6 DE lncRNAs with 29 genes from the IPA knowledgebase (enrichment score of 14; Fig. 2c). Network scores are based on the network-eligible molecules in the analysis. Scores are inversely related to the probability of finding the selected network-eligible molecules in a given network by random chance.

**Table 2:** Biological functions from IPA knowledgebase that are enriched with DE lncRNAs

Disease and Biofunctions	P-values	Molecules
Breast or colorectal cancer	0.0049	C10orf25, CASC2, CRNDE, FOXD2-AS1, HCG11, HOTAIRM1, LINC00346, NORAD, PANDAR
Development of digestive organ tumor	0.0122	C10orf25, CASC2, CRNDE, EPB41L4A-AS1, FOXD2-AS1, HOTAIRM1, PCA3, PVT1
Development of carcinoma	0.0176	C10orf25, CASC2, CRNDE, CYTOR, EPB41L4A-AS1, FOXD2-AS1, HOTAIRM1, PCA3, PVT1
Colorectal carcinoma	0.0231	C10orf25, CRNDE, FOXD2-AS1, HOTAIRM1
Malignant genitourinary solid tumor	0.0254	CASC15, CASC2, CRNDE, HCG11, LINC00346, NORAD, PANDAR, PCA3

The p-values are calculated with Fisher exact test. Molecules listed here are those DE lncRNAs identified within the functional group being assessed

### Nomination of lncRNA as a miRNA sponge

**Fig. 3:**
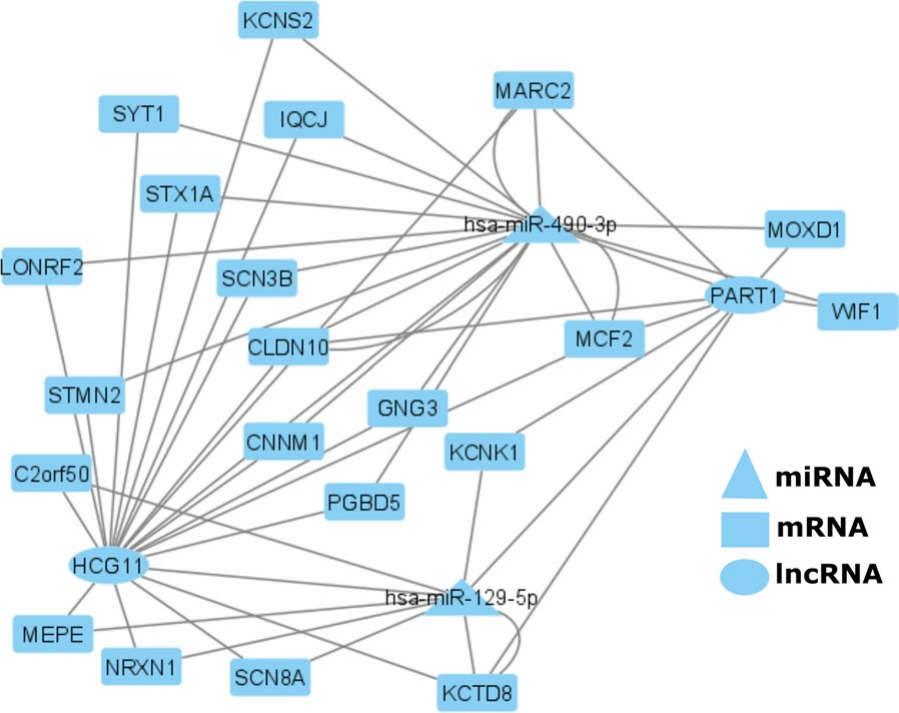
The network of sponge interaction between HCG11, PART-1, hsa-miR-129-5p, hsa-miR-490-3p, and mRNAs (IPA knowledgebase was used to restrict the figure to known mRNA targets for these 2 miRNAs). The network was drawn using the Cytoscape (v3.4.0) [35]

Since less than 10% of the lncRNAs had known function in the pathway analysis we also used a data driven approach to identify lncRNA functioning as miRNA sponges. To identify lncRNA:miRNA:mRNA triplets (sponge relationships), Spearman rank order correlation (r_x,y_) was estimated on expression levels between each mRNA and DE lncRNA. As described in the methods, the r_x,y_ > 0.5 threshold resulted in 580 (lncRNAs) and 14,425 (mRNAs) selected. After filtering the correlated pairs to only those with a common miRNA target for the lncRNA and mRNA, 121,276 triplets were constructed from 15 lncRNAs, 6777 mRNAs, and 201 miRNAs. To assess if a triplet was likely to reflect a sponge relationship, the partial correlation (r_x,y|z_) between each lncRNA and the correlated mRNA was estimated, controlling for the predicted common miRNA. The distribution of the influence of miRNA on the lncRNA:mRNA correlation, specifically Sz = r_x,y_—r_x,y|z_, is plotted in Additional file 1: Fig S1. Two miRNAs fell into the 99th percentile of this Sz distribution: hsa-miR-129-5p, hsa-miR-490-3p. Associated with these two miRNAs were two lncRNAs (HCG11, PART1), which were correlated with 290 (HCG11) and 114 (PART1) mRNAs, respectively, forming miRNA:lncRNA:mRNA trios. Thus, HCG11 and PART1 were nominated as sponges for hsa-miR-129-5p and hsa-miR-490-3p, blocking their interaction with 342 unique mRNA; Fig. 3.

## Discussion

In our investigation of the non-coding transcriptome between G-CIMP-high and G-CMIP-low glioma subclasses, 666 differential lncRNAs were identified; some of these lncRNA had previously been associated with oncogenic activities in cancer. The expression levels of the majority of these lncRNAs were lower in G-CIMP-high tumors. The up-regulation in G-CIMP-low tumors may relate to oncogenic activities leading to their aggressive phenotype and poor survival. A previously known relationship between TP53 (p53) and PVT1, as a p53-induced target gene, was observed by examination of upstream regulators of the differential lncRNA [13]. Further, we nominated two lncRNAs as having potential sponge activity for two microRNAs.

### TP53 as a regulator of PANDAR and PVT1

TP53 is a well-known gene for a tumor suppressor protein p53 that participates in diverse cellular functions including cell cycle arrest, apoptosis, senescence, DNA repair, and changes in metabolism. Mutation of TP53 is associated with a variety of human cancers including gliomas, and is found in 94 percent of IDH mutant, 1p/19q non-codeleted, glioma [14]. High expression of PVT1, a long non-coding RNA located at chromosome 8a24.21, has been associated with several mutations of TP53 in diffuse glioma [8]. In these data, the expression of TP53 and PVT1 are positively correlated, with highest expression of each occurring in GCIMP-low, see Additional file 2: Fig S2. Many studies have shown evidence of carcinogenic activity of PVT1 in various cancers, such as negatively modulating miRNA by acting as a competing endogenous RNA or acting as a sponge to promote tumor effects [15]. Of importance to glioma, PVT1 has been implicated in regulating levels the proto-oncogene MYC to promote tumorigenesis [16]. The role of MYC in glioma has been well established, both in vivo and in vitro, such that MYC inhibition suppresses glioma formation, restricts glioma cell proliferation and improves survival [17]. The relationship between TP53 and PANDAR is less understood, though human p53 [TP53] protein is necessary for expression of human PANDA [PANDAR] lncRNA. PANDAR is a promoter of CDKN1A antisense DNA damage activated RNA and increased expression of PANDAR has been indicated to predict poor prognosis in cervical and gastric cancer[18, 19]. Recently, a study published showed CDKN2A, a gene which belongs to same family as CDKN1A, often deleted in G-CIMP-low tumors as compared to G-CIMP-high [20]. While the role of PANDAR has been evaluated in many cancers [21] its association with glioma has not been studied.

### Network focused on cell growth, proliferation, survival and death

Cell death is one of the primary mechanisms studied in cancer as disruption of this process can facilitate tumorigenesis, promote proliferation, and lead to resistance to anticancer therapy. One of the top gene–gene networks generated from the IPA knowledgebase was associated with the biological processes of cell death, survival, cell growth and proliferation (Fig. 2c). This network was constructed from 6 DE lncRNAs (PVT1, PANDAR, FOXD2-AS1, CYTOR CRNDE, HOTAIRM1) and 29 mRNAs that interact directly (solid lines) or indirectly via an intermediate gene (dotted line). Among the interacting mRNAs, the NOTCH1 gene’s role in glioma pathogenesis is well established as it affects glioma tumorigenesis and maintenance. Several studies during recent years reported dysregulated NOTCH signaling activity (NOTCH 1–4) in human brain tumors [22]. In an extensive study on the functional role of NOTCH1 in gliomas, it is observed that NOTCH1 is involved in maintaining glioma cells in an undifferentiated state, and its inhibition leads to cells maturing into a less aggressive phenotype [22]. Also, in the network (Fig. 2c) is MYC, a proto-oncogene that encodes nuclear phosphoprotein control as a transcription factor for its target genes. As described above, we see again the association between MYC and the PANDAR lncRNA.

### Enrichment of oncogenic function

The biofunction “development of carcinoma” was one of the top cancer-related enriched biofunctions (p-value of 0.0176), with 8 DE lncRNAs: PVT1, CASC2, PCA3, EPB41L4A-AS1, C10orf25, CYTOR, FOXD2-AS1, and CRNDE. The increased expression of PVT1, CYTOR, FOXD2-AS1, and CRNDE were seen in various cancers, similarly these lncRNAs were all up-regulated in G-CIMP-low suggesting their more oncogenic activity leads to poor prognosis compared to G-CIMP-high. While decreased expression has been seen in CASC2 and PCA3 in cancer, these lncRNAs were down-regulated in G-CIMP-low suggesting their tumor suppressor potential [23–27]. Table 2 shows other carcinogenic functions from the enrichment of DE lncRNAs with IPA knowledgebase.

### Nominated lncRNA sponge activity

Research in many cancers has shown that lncRNAs can regulate mRNA expression levels indirectly through miRNA, by acting as a miRNA sponge. LncRNAs HCG11 and PART-1 were identified as potential sponges for the miRNAs hsa-miR-490-3p and hsa-miR-129-5p (Fig. 3). Previous studies [28] have shown lncRNA HCG11 to be down-regulated in glioma tissues and cells, and this was associated with a lower survival rate in glioma patients. The observed mechanism is for lncRNA HCG11 to suppress growth of glioma was by acting with the miR-4425 to release MTA3. MiR-4425 is up-regulated in glioma tissues and a high expression of miR-4425 is associated with an unfavorable prognosis in glioma [28]. Here we also see decreased expression of HCG11 in our lower survival G-CIMP-low group and propose an interaction with miR-490 and miR-129. LncRNA PART-1 has been shown to have oncogenic activity in colorectal cancer [29], but was identified as positively associated with GBM prognosis [30], such that decreased PART-1 predicted decreased survival time [31]. In this study we observed that PART-1 expression was lower in the poor-prognosis G-CIMP-low tumors, compared to G-CIMP-high tumors, which aligns with the observed relationship in GBM. Neither lncRNA HCG11 nor PART1 have been investigated for a relationship with hsa-miR-129-5p and hsa-miR-490-3p in glioma. From prior research in lung and hepatocellular carcinoma, increased hsa-miR-490-3p has been implicated in cell migration and cancer progression to metastatic disease [32, 33]. Reduction of hsa-miR-490-3p through sponge action of lncRNAs suggests a more favorable outcome, which we see in G-CMIP-high. In contrast, hsa-miR-129-5p has been shown to inhibit the cell cycle and induce apoptosis in glioma cell lines through inhibition of NOTCH1 and mTOR signaling [34]. Reduction of has-miR-129-5p through sponge action may thus allow increased proliferation, which is counter to expectation but dependent upon signaling pathways, so more study is needed.

## Conclusion

This in-silico study explores the potential influence of non-coding RNA on the phenotypic difference between the G-CMIP-high and G-CMIP-low subtypes of glioma. The G-CMIP-low subtype is rare in primary glioma diagnosis, with less than 5% of all diffuse glioma diagnoses identified as G-CMIP-low. However, prior work shows that G-CMIP-high tumors may evolve to a G-CMIP-low form as the disease progresses [36]. With this in-silico study, we identified 666 lncRNAs that showed a difference in mean expression between the two G-CMIP subtypes. With the IPA knowledgebase, we were able to propose the functional role of a subset of differential lncRNAs related to progression to aggressive G-CIMP-low gliomas. In addition, we identified an upstream regulator, TP53, a well-known tumor suppressor gene which can regulate two of the differential lncRNAs. Unfortunately, our study is not without limitation, as only 44 of 666 lncRNAs had biological function information in the IPA knowledgebase. We were heartened that these 44 showed oncogenic relationships with genes known to have a role in glioma, however, we realize that there is much to be discovered among 622 lncRNAs with no information in IPA. Beyond IPA, we also identified two lncRNAs as potentially having miR-sponge activity, HCG11 and PART-1. Each has prior evidence of an effect on glioma prognosis, which increases our enthusiasm for further study.

## Methods

### Ethics statement

RNA-sequencing datafiles (TCGA Glioblastoma (GBMs) and Lower-grade glioma (LGGs)) were obtained from the Genomic Data Commons with appropriate approval from dbGAP (#1904). They were acquired with a protocol approved by the Henry Ford Health System institutional review board (protocol #8718). The need for consent was waived in this secondary data analysis since primary identifiers were not provided by dbGAP.

### Quantification of mRNA & lncRNAs

Aligned sequencing reads (BAM files) for TCGA GBMs and LGGs, generated from the Illumina Hiseq platform, were obtained from the Genomic Data Commons database (GDC) (March 2017). These Illumina raw reads had been processed through the RNA-Seq standardized pipeline at GDC. Briefly, the GDC pipeline first converted to fastq with ‘Biobambam’ and then re-aligned to the GRCh38 reference genome per alignment guidelines from International Cancer Genome Consortium (ICGC), using the STAR aligner. A two-pass method was used for alignment; first, splice-junctions were aligned separately in each read group, then the read groups were merged to obtain the final alignment in BAM format. Upon downloading these aligned BAM files, we quantified the read counts for lncRNAs and protein-coding messenger RNA (mRNA) against the reference annotation from GENCODE v28 [37]. This quantification was executed with the ‘featurecount’ function, from the R Bioconductor package ‘Rsubread,’ [38] which assigns mapped sequencing reads to genomic features. Two-hundred fifty samples (see Table 1) and 37,281 features (22,583 mRNAs and 5729 antisense, 7845 lincRNA, 939 sense-intronic, 185 sense-overlapping lncRNA) were carried forward for further analysis.

### Identifying differentially expressed sets of mRNA & lncRNAs specific to subtypes

The quantified expression matrix (in terms of read counts) for each sample was further filtered for low count, based on counts per million (CPM). Features with sum of expression values below the condition cut-off (CPM < 1) across conditions were removed. Normalization between cases was performed on the weighted trimmed mean of the log2 expression ratios (TMM; trimmed means of M-values) using the R Bioconductor package NOISeqBio [39]. This normalization method assumes that the majority of the RNA features are not differential. After pre-processing, 24,178 features were retained for analysis. The NOISeqBIO package was used to identify differential expression per feature (mRNA and lncRNA) between G-CIMP-high and G-CIMP-low, considering the log2-ratio of the two conditions (M-value) and the value of the difference between conditions (D-value). A feature was identified as differential between conditions if its corresponding M and D values are likely to be higher than the expected noise, where the noise distribution is obtained from comparing all sample pairs within a condition. lncRNA were identified as differential between the two groups if the false discovery rate (FDR) was less than or equal to 5%. The differential mRNA and lncRNA identified in this analysis are presented in Additional files 3 and 4.

### Pathway analysis of differential lncRNA

LncRNAs that were differentially expressed between G-CIMP-high and G-CIMP-low tumors were further evaluated for biological functional interpretation with Ingenuity’s IPA software knowledgebase [40]. The core enrichment analysis was performed using all lncRNAs from the Gencodev28 annotation, described above, as the reference set for the Fisher’s exact test used to calculate enrichment p-values.

### Integrative analysis of lncRNAs, miRNAs and mRNA to predict miRNA sponge activity

In scenarios where a lncRNA is acting as miRNA sponge, it is expected that the correlation between lncRNA and mRNA expression will be positive. Further, the correlation between lncRNA and mRNA will be dependent on miRNA expression, such that it lessens when the miRNA expression is considered. For this analysis, TCGA miRNA-seq data were downloaded from the Broad Firehose for 239 samples (G-CIMP-high = 228, G-CIMP-low = 11). Data had been aligned and quantified by Broad, reporting log2 reads per million (RPM) for 2588 miRNAs. Transcriptome-wide microRNA target prediction for each lncRNA and mRNA observed to be differentially expressed between G-CIMP-high and G-CIMP-low was obtained from MiRcode [41] annotation. Correlated lncRNA and mRNA pairs with a common miRNA target were retained for further analysis. To assess whether a sponge relationship is likely within each lncRNA:mRNA:miRNA trio, correlation between the lncRNA and mRNA expression was estimated, alone (r_x,y_; Spearman rank order correlation) and controlling for the target miRNA expression (r_x,y|z_; Spearman rank order partial correlation) [42]. The unconditional correlation was filtered to r_x,y_ >  = 0.5, with p-value <  = 0.05. Then a lncRNA was nominated as having a sponge function in the lncRNA:miRNA:mRNA trio if Sz = r_x,y_—r_x,y|z_ was high; here we use Sz > 0.2 [42].

## Supplementary Information

**Additional file 1: Fig. S1.** The distribution of the influence of miRNA on the lncRNA:mRNA correlation, Sz = r_x,y_—r_x,y|z_, is plotted. For RNA triplets a reduction of correlation (S_z_) of 0.2 or great were retained, in this study 0.2 is approximately the 99th percentile of the distribution of the S_z_ distribution.

**Additional file 2: Fig S2.** Scatterplot of the expression of TP53 and PVT1 in G-CIMP-high and G-CIMP-low tumors.

**Additional file 3: Table S1.** Differential_LncRNAs.

**Additional file 4: Table S2.** Differential_mRNAs.

## Data Availability

RNA-sequencing datafiles (TCGA Glioblastoma (GBMs) and Lower-grade glioma (LGGs)) were obtained from the Genomic Data Commons with appropriate approval from dbGAP (#1904). Results datasets supporting the conclusions of this article are included in the additional files.
