# Supplementary figures and images for: Expression and regulatory roles of lncRNAs in G-CIMP-low vs G-CIMP-high Glioma: an in-silico analysis
Source: J Transl Med. 2021 Apr 29;19:182. doi: 10.1186/s12967-021-02844-z (PMC8086286; doi:10.1186/s12967-021-02844-z)

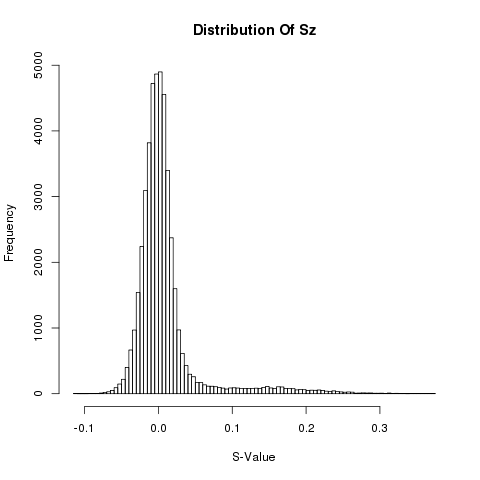

Supplement: Supplementary file 1 — Additional file 1: Fig. S1. The distribution of the influence of miRNA on the lncRNA:mRNA correlation, Sz = rx,y—rx,y|z, is plotted. For RNA triplets a reduction of correlation (Sz) of 0.2 or great were retained, in this study 0.2 is approximately the 99th percentile of the distribution of the Sz distribution. [file 12967_2021_2844_MOESM1_ESM.png]

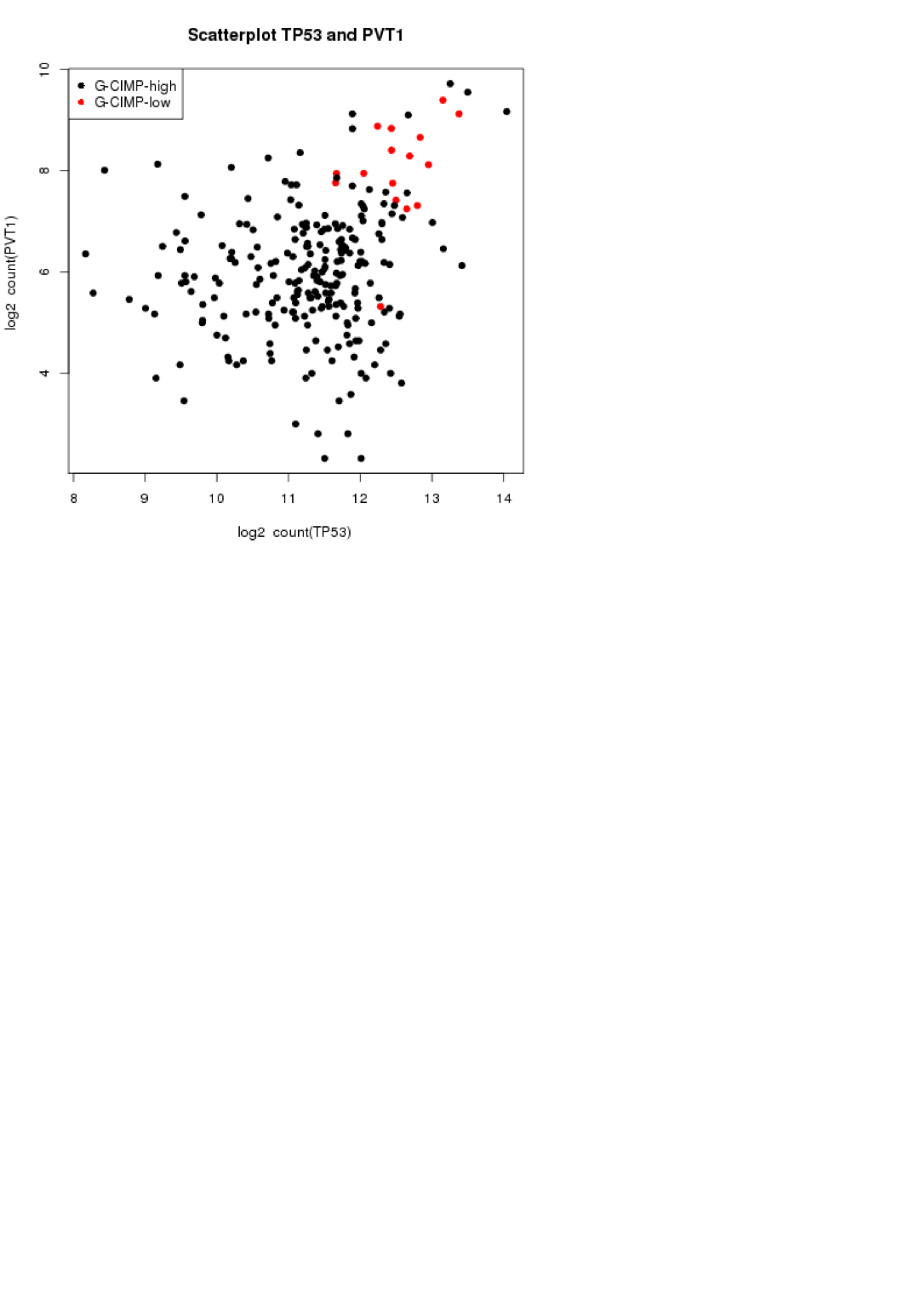

Supplement: Supplementary file 2 — Additional file 2: Fig S2. Scatterplot of the expression of TP53 and PVT1 in G-CIMP-high and G-CIMP-low tumors. [file 12967_2021_2844_MOESM2_ESM.png]
